# Conventional Chemotherapy and Inflammation: What Is the Role of the Inflammasome in the Tumor Microenvironment?

**DOI:** 10.3390/biomedicines13010203

**Published:** 2025-01-15

**Authors:** Chiara Colarusso, Michela Terlizzi, Simone Di Caprio, Anna Falanga, Emmanuel D’Andria, Roberta d’Emmanuele di Villa Bianca, Rosalinda Sorrentino

**Affiliations:** 1 Department of Pharmacy (DIFARMA), University of Salerno, 84084 Fisciano, SA, Italy; ccolarusso@unisa.it (C.C.); mterlizzi@unisa.it (M.T.); sdicaprio@unisa.it (S.D.C.); afalanga@unisa.it (A.F.); emmdandria@unisa.it (E.D.); 2 Department of Pharmacy, University of Naples Federico II, 80131 Naples, NA, Italy; roberta.demmanueledivillabianca@unina.it

**Keywords:** cancer, chemotherapy, inflammasome, immunogenic-cell death (ICD), inflammation

## Abstract

The link between inflammation and cancer has been extensively studied over the years. While the inflammatory process can facilitate tumor establishment and progression, on the other hand, current clinical approaches aim to boost the immune system against the tumor mass. In this scenario, the conventional chemotherapy has proven to induce immunogenic cell death in that the release of danger-associated alarmins can foster the cytotoxic immunity following the blockade of immune checkpoints. The release of alarmins can activate the inflammasome pathway. Thus, one of the questions is as follows: can conventional anti-tumor drugs lead to inflammasome activation? And if so, is the resulting effect anti- or pro-tumor? In this review, we provide an overview on the role of the inflammasome in cancer.

## 1. Introduction

The link between inflammation and cancer has been studied for years, and although many advances have been made, the age-old question about the chicken or the egg reflects the precise mechanism that correlates inflammation to cancer and vice versa. In this context, the role of the immune system before and after pharmacological treatment uncovers a very important role in that if appropriately activated, it can provide an anti-tumor activity.

Inflammation is a physiological response that protects the host against pathogen-associated molecular patterns (PAMPs) and damage-associated molecular patterns (DAMPs). In both cases, inflamed/damaged tissues represent the alarm to restore physiological homeostasis. In the context of a neoplastic transformation, the inflammatory process, in the attempt to eliminate the endogenous danger signal, can facilitate tumor establishment and progression [1]. This scenario occurs especially when anti-inflammatory/remodeling pathways are established. The physiological downmodulation of the immune system once the antigen is recognized can similarly occur in the tumor microenvironment (TME), facilitating tumor immune escape [2,3,4].

The classification of tumors as “hot” or “cold” [5] has established the concept of the *immunoscore*, which, according to the phenotype of the immune cells, can predict patients’ prognosis and drug responsiveness. In this regard, a cold tumor could be rendered hot by using classical chemotherapeutics, which can induce tumor cell death, the so-called immunogenic cell death (ICD) [6], with the generation/release of alarmins that can activate pathogen recognition receptors (PRRs). The double-edged sword is that the inflammatory response can be counteracted by downmodulating the immune reaction, an immunosuppressive TME ensuing.

In this review, we intend to evaluate the role of the inflammasome after chemotherapeutic treatment. The inflammasome, a multiprotein complex responsible for IL-1-like cytokine release, is a critical component of the innate immune response, the deregulation of which can lead to cancer initiation and progression [7,8,9]. The inflammasome complex comprises the assembly of NOD-like receptors (NLRs) or hematopoietic interferon-inducible nuclear proteins with 200-amino-acid repeat (HIN200) family receptors, such as absent in melanoma 2 (AIM2); is able to bind the adaptor apoptosis-associated speck-like protein (ASC), which acts as a ‘zipper’ protein, ensuing pro-caspase-1 auto-cleavage, which in its active form facilitates the activation and then the release of IL-1-like cytokines [10]. Although several types of inflammasomes have been identified both in humans and mice, the inflammasome nucleotide-binding domain leucine-rich-containing family pyrin domain-containing-3 (NLRP3), a NLRs that contain a C-terminal leucine-rich repeat (LRR) domain, a central nucleotide-binding domain (NBD), Apaf-1, CIITA, HET-E, and a TP1 (NACHT), and an N-terminal pyrin domain (PYR), are the best characterized. It was reported that the NLRP3 inflammasome pathway can be broadly divided into two signal modes, priming and activation. The priming step involves the expression of the inflammasome components via Toll-like receptor (TLR)-, transcription factor-, and posttranslational modification-mediated signaling, whereas the activation step involves the oligomerization of the sensor (e.g., NLRP3), the adaptor ASC, and the effector pro-caspase-1 to activate the entire inflammasome complex according to the exogenous/endogenous stimulus [11]. Additionally, the activation of the NLRP3 inflammasome can also occur after the interferon (IFN)-dependent signaling pathway. In particular, the activation of NLRP3 in dendritic cells (DCs) showed an anti-tumor activity via IL-1β/Th1/IFNγ [12].

Based on the concept that many chemotherapeutic agents (gemcitabine, 5-fluorouracil (5-FU), doxorubicin, vincristine, melphalan, cisplatin, etoposide, paclitaxel, and methotrexate) have been shown to activate the inflammasome complex [13,14,15,16], the main questions here are as follows: Can conventional anti-tumor drugs lead to the inflammasome activation? If so, is the resulting effect anti- or pro-tumor? Therefore, here, we provide an overview of the role of the inflammasome after treatment with classical chemotherapy in order to understand whether therapy-induced immune system activation can interfere with the growth of residual malignant cells or whether it improves the therapeutic outcome based on the inflammasome activation. To this aim, we reviewed the recent findings published in the last decade in this field by performing a literature search using PubMed and exploring for different keywords, such as “inflammasome and cancer”, “inflammasome activation and chemotherapy”, and “inflammasome and immunogenic-cell death (ICD)”.

## 2. Immunogenic Cell Death (ICD)

The success of an anti-cancer therapeutic strategy mainly depends on the ability to induce cancer cells to death, promoting tumor regression or stability. The main mechanisms by which a malignant cell can die are apoptosis, necrosis, pyroptosis, and autophagy [17], all mechanisms of ICD that elicit an immune response due to the release of cancer cell-derived DAMPs.

The concept of ICD was originally proposed in 2005 by Casares, who stated that “dying cancer cells emit a spatiotemporally defined combination of signal/s that, at least under selected circumstances, can be translated by the immune system into an anti-tumor response” [18]. Therefore, the administration of conventional anti-tumor agents, whose molecular mechanism is to promote ICD, can lead to the release or identification of dying cell-derived DAMPs by specific molecular patterns. The release of DAMPs after chemotherapy is mediated by the endoplasmic reticulum (ER) stress and ensuing generation of reactive forms of molecular oxygen (i.e., reactive oxygen species, ROS), crucial for the induction of a specific humoral or cellular immune response via the activation of the inflammasome complex. So far, tumor-derived or chemotherapy-induced ICD with an ensuing release of alarmins is able to activate the NLRP3 inflammasome due to the alteration of the mitochondrial homeostasis [10] or the AIM2 inflammasome after the recognition of double-stranded DNA (dsDNA) released by dying cells [19,20,21,22,23,24]. Therefore, the question arises spontaneously: if the release of DAMPs induced by the chemotherapy treatment can activate the inflammasome, what role does this complex play in terms of tumor regression or progression? Since the inflammasome has a controversial role in cancer, what happens after treatment with chemotherapy or after the combination of chemotherapy and immune checkpoint inhibitors (ICIs)? In the following paragraphs, we discuss the activation of the inflammasome following the release of tumor-derived alarmins.

### 2.1. ICD-Associated DAMPs and Inflammasome Activation

Adenosine Triphosphate (ATP) and High Mobility Group Box 1 (HMGB1) are DAMPs secreted or released by tumor cells, whereas others, such as calreticulin (CRT) and heat shock protein 90 (HSP90), are exposed de novo or overexpressed by both tumor and immune cells. Most of these molecules have predominantly non-immunological functions inside the cell before their exposure on the cell surface or their secretion [25].

#### 2.1.1. ATP

**Figure 1:**
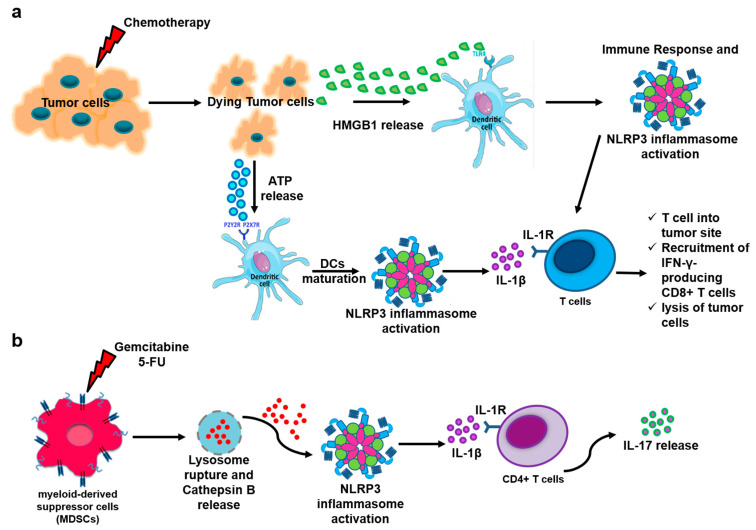
Cell death mechanisms. (**a**) Chemotherapy can induce immunogenic cell death (ICD) via the release of Adenosine Triphosphate (ATP) and High Mobility Group Box 1 (HMGB1) as well as the exposure of alarmins on the membrane of tumor cells in that specific immunogenicity is induced with an ensuing attack of the tumor mass by the immune system. Specifically, on one side, ATP released by dying tumor cells binds the P2X7 receptor (P2X7R) on the dendritic cell (DC) surface, favoring DC maturation and nucleotide-binding domain, leucine-rich-containing family, pyrin domain-containing-3 (NLRP3) inflammasome-dependent IL-1β release, which together with antigen presentation of tumor-associated antigens, induces the recruitment of T cells into the tumor mass. In contrast, dying tumor cells release HMGB1, which may be recognized by Toll-like receptor 4 (TLR4)/myeloid differentiation primary response gene 88 (MyD88) pathway, leading to a potent immune response. (**b**) Gemcitabine and 5-fluorouracil (5-FU) can induce the activation of NLRP3 via lysosome rupture and cathepsin B release, enhancing an IL-1β-dependent T helper 17 (Th17) response.

It is widely believed that ATP released by dying cancer cells is required for inducing an anti-tumor immune response (Figure 1a). Extracellular ATP binds P2Y2 receptors (P2Y2R) on monocytes and P2X7 receptor (P2X7R) on dendritic cells (DCs) [26,27], allowing for DC maturation with an overexpression of costimulatory molecules, such as CD40, CD80, and CD86 [28]. More importantly, ATP released from dying cancer cells can bind P2X7R on DCs and induce the NLRP3 inflammasome activation and secretion of IL-1β, which together with antigen presentation of tumor-associated antigens (TAA), leads to the polarization of interferon-γ (IFN-γ)-producing CD8^+^ T cells, which can counteract cancer cell survival/proliferation [27] (Figure 1a). Thus, ATP-induced inflammasome activation may be crucial for the success of an anti-cancer therapy, supported by the fact that *P2X7R* knockout mice as well as mice deficient in inflammasome components (i.e., NLRP3, ASC, and caspase-1) or Interleukin 1 receptor type I (IL-1R1) are refractory to chemotherapy [29]. This implies that the extracellular ATP can exert an anti-tumor response induced by chemotherapy [29]. In sharp contrast, recent findings suggest that P2X7R antagonists could suppress cancer progression by altering tumor cell context. It was found that pancreatic ductal adenocarcinoma (PDAC) cells treated with a P2X7R allosteric inhibitor showed reduced tumor proliferation and invasion compared to untreated cells [30]. Moreover, P2X7R inhibition can lead to attenuated invasiveness in epidermoid carcinoma-derived cells A253 [31]. In support of the potential use of antagonists of P2X7R pathways in cancer therapy, a phase I study demonstrated that approximately 65% of patients with basal cell carcinoma showed a decrease in lesion areas [32]. Importantly, according to the low half-life of ATP, which is rapidly converted into adenosine via the activation of CD39 and CD73 and highly expressed on regulatory T cells (T regs) and CD8^+^-exhausted T cells, it is likely that this TME facilitates the immunosuppressive adaptive immunity in that the accumulation of T regs and regulatory B cells (B regs) can support tumor cell proliferation rather than eradication, as observed for lung and breast tumor models [33].

#### 2.1.2. HMGB1

HMGB1, released by dying tumor cells, may be recognized by DCs in a Toll-like receptor 4 (TLR4)- and myeloid differentiation primary response gene 88 (MyD88)-dependent manner, leading to a potent immune response (Figure 1a). Because TLR4 is involved in the two-signal model of the NLRP3 inflammasome activation, it is tentative to think that its activity after chemotherapeutic treatment can be protective, as demonstrated in a mouse model of colon cancer. Mice deficient in NLRP3 had a lower survival rate than the wild type in that they were less prone to apoptosis in favor of tumor cell proliferation [34]. Nevertheless, HMGB1 can have a dual effect in that it can be released as an alarmin, activate an immune response, and participate in the regulation of inflammation and cancer progression [35]. In support, it was also demonstrated that colon [36] and lung [37] cancers were exacerbated in a TLR4-dependent manner, implying that tissue specificity and homeostasis represent the rheostat for cell proliferation vs. cell death.

#### 2.1.3. CRT

In the early course of ICD, after ER stress, CRT relocates to the plasma membrane favoring recognition and phagocytosis by macrophages and DCs, inducing an anti-tumor immune response [28]. In response to specific chemotherapeutics (such as anthracyclines), CRT facilitates the engulfment of dying cancer cells by DCs resulting in tumor antigen presentation and tumor-specific cytotoxic T lymphocyte (CTL) responses [38]. In particular, CRT exposed on tumor cells undergoing ICD are phagocyted by immature DCs, that migrating to the lymph nodes cross-present to CD8^+^ T cells eliciting a strong anti-tumor activity. Another study demonstrated that CRT was involved in the NLRP3 activation signal [39], most likely via the alteration of Ca^2+^ homeostasis in tumor cells. Since the alteration of Ca^2+^ levels can also induce NLRP3 activation, it is likely that the whole complex can participate to resistance to chemotherapy (tumor progression) via the activation of the inflammasome. To note, high CRT gene (*CALR*) transcription has been correlated with rapid tumor progression and poor prognosis of patients affected by gastric malignancy, Non-Small Cell Lung Cancer (NSCLC), breast carcinoma, neuroblastoma, pancreatic cancer, bladder carcinoma, and mantle cell lymphoma [40]. These findings suggest that high levels of CRT may be required for some tumors to progress.

#### 2.1.4. HSP90

HSP90 is normally located in the intracellular compartment, and its release or surface expression offers a direct immunogenic signal for immune system activation (especially DC activation) [28]. HSP90 can form a complex with NLRP3, retaining it in an inactive but competent form; thus, in the presence of HSP90, NLRP3 can undergo degradation by the proteasome [41]. According to this last concept, various HSP90 inhibitors, which indirectly block NLRP3 priming and activation, have been proposed in the treatment of inflammasome-dependent diseases [41], obtaining promising results, especially in the treatment of solid tumors [42]. However, resistance to HSP90 inhibitors has been demonstrated as the major reason for their limited effectiveness as monotherapy [43], paving the way to the investigation of combination therapy strategies. In this context, it was found that the administration of the HSP90 inhibitor AUY922 in combination with doxorubicin resulted in increased levels of caspase-3 expression, a biomarker of mitochondrial apoptosis, and decreased levels of vascular endothelial growth factor (VEGF) mRNA, an effect that was not observed in monotherapy treatments [44].

#### 2.1.5. Self-DNA

Self-DNA, widely studied in autoimmune disorders, is secreted during chemotherapy-induced ICD. It was reported that chemotherapeutic agents, such as cisplatin, etoposide, or radiation therapy, are able to induce innate immune responses, leading to the release of self-DNA from tumor dying cells [45]. The release of self-DNA is able to access the cytosol of DCs to induce the secretion of cytokines by the activation of the cyclic GMP-AMP synthase (cGAS)/stimulator of interferon gene (STING) pathway (cancer development ensuing [45]). The opposite effect was observed for T cell activation that exerted anti-tumor activities [46]. However, tumor-derived DNA can also be recognized by the AIM2 receptor, which was described at the interface between chronic lung inflammation, as in the case of chronic obstructive pulmonary disease (COPD) and NSCLC [21]. High levels of AIM2 in the tumor mass were associated with poor prognosis of NSCLC patients, defining two types of inflammatory profiles, which are inflammasome and non-inflammasome dependent [23].

## 3. Activation of the Inflammasome After Chemotherapy-Induced ICD: Tumor Growth or Arrest?

Many studies have been performed on the role of chemotherapeutics and their efficacy in cancer. ICD induced by conventional chemotherapy has raised questions regarding the role of the inflammasome at recognizing tumor-derived DAMPs. Therefore, beyond the nature of released alarmin, the activation of the inflammasome seems to be at the interplay between pro- or anti-cancer activity.

For instance, the activation of the NLRP3 inflammasome after anthracycline treatment resulted in anti-tumor activity [47] (Table 1), whereas the administration of paclitaxel, 5-FU, or gemcitabine had opposite effects [47]. Thus, the literature shows discrepant data on what happens downstream from the activation of the inflammasome in terms of tumor cell growth. Most likely, the answer may depend on the type of tumor and microenvironment as well as the immune infiltration (*immunoscore*). In support, the combination of paclitaxel [48] and Immunotherapeutics (such as pembrolizumab in NSCLC), proved anti-tumor efficacy in that both progression-free survival (PFS) and overall survival (OS) are ameliorated [49,50,51]. This concept is nowadays accepted, especially for oncological patients who have a low/intermediate (<49%) expression of Programmed Death-Ligand 1 (PD-L1). The combination of chemotherapy with immunotherapy (especially anti-PD-L1 antibodies) has found ground in the clinic because it showed a better PFS and OS rate [52]. To note, besides clinical immunotherapy in combination or not with conventional chemotherapy, pharmacoepidemiologic studies report almost 30% drug resistance due to both innate and acquired mechanism/s [53].

Inflammasome-targeting chemotherapeutic agents and their effects are summarized in Table 1.

### The Blockade of the Inflammasome Can Enhance Chemotherapy Efficacy

It was demonstrated that paclitaxel mediates NLRP3 inflammasome priming via ATP release attenuating its chemotherapeutic potential [14]. In this study, paclitaxel/ATP release induced the inflammasome activation via TLR4 that led to the phosphorylation of IkB and Jun N-terminal kinase (JNK) and the upregulation of pro-inflammatory cytokines [14] in favor of tumor growth.

The administration of gemcitabine and 5-FU on MSC-2 cells, an established myeloid-derived suppressor (MDSC) cell line, led to the activation of NLRP3 via lysosome permeabilization and cathepsin B release enhancing an IL-1β-dependent T helper 17 (Th17) response, which limited the anti-cancer efficacy due to a proangiogenic effect [54] (Figure 1b, Table 1). Instead, the same authors previously demonstrated that anthracycline-induced ICD is associated with the release of IL-1β and priming of anti-cancer CD8^+^ T cells [55]. These discrepant results could underlie the fact that gemcitabine and 5-FU do not induce immunogenic tumor cell death; consequently, these drugs do not favor the cross presentation of tumor antigens by DCs and CD8^+^ T cell activation [56].

Feng and colleagues found that the NLRP3/IL-1β axis favored 5-FU resistance of oral squamous cell carcinoma both in vitro and in vivo [57]. Their findings highlighted that NLRP3 expression and activation were higher in cancerous tissues from 5-FU-treated patients, whose prognosis was poor according to the tumor stage and differentiation [57].

In support, in 2006, the US Food and Drug Administration (FDA) authorized thalidomide for malignant myeloma since it can inhibit caspase-1 [58] and AIM2 inflammasome [59]. In a randomized phase II study evaluating the combination of thalidomide and docetaxel in patients with metastatic androgen-independent prostate cancer, a significant decrease of the prostatic-specific antigen (PSA) levels and an increase in the median survival rate of patients was found, pointing at inflammasome inhibition as a potential mechanism of efficacy of docetaxel [60].

Furthermore, a very interesting study found that chimeric antigen receptor T cell (CAR-T) therapy, combined with the blockade of the AIM2 inflammasome or α1-adrenergic receptor (α1-AR), involved in both AIM2 activation and overexpression, may relieve IL-1β-related toxic side effects of CAR-T therapy and ensure anti-tumor effects of the treatment [61]. In particular, the cooperation of the AIM2 inflammasome pathway and adrenergic-signaling-induced macrophage polarization towards an immunosuppressive phenotype by upregulating the expression of PD-L1 and indoleamine 2,3-dioxygenase (IDO) [61]. Therefore, blocking AIM2 inflammasome or α1-AR reduces the production of bioactive IL-1β and reverses macrophage phenotype triggered by CAR-T therapy, thus enhancing an anti-tumor effect.

In support, the pro-tumor activity of the inflammasome can be proven by the blockade of IL-1β using Anakinra, which proved to prolong the PFS of myeloma patients through the reduction of IL-6 levels, crucial for tumor cell proliferation and survival [62]. It was found that Anakinra administration repressed cell proliferation and angiogenesis, but it was unsuccessful in augmenting cancer cell death in mouse models of breast cancer [63], implying that the inhibition of the cytokine alone is not as efficient as if it was associated with other anti-tumor treatments. In 2009, Mayo Clinic started a phase II clinical trial that showed that Anakinra targets the progressed myeloma fraction in vivo and decreases the proliferation of myeloma cells [64]. In a phase II study, the therapy with Anakinra plus 5-FU and bevacizumab (an anti-VEGF agent) revealed an impressive survival rate of patients with metastatic colorectal cancer, with a median PFS of 5.4 months and OS of 14.5 months [65]. These data suggest that the coadministration of 5-FU/bevacizumab/Anakinra might become a potential treatment option for patients with refractory metastatic colorectal cancer and that therapy targeting inflammasome stimulation could be useful to improve the efficacy of adjuvant or palliative cancer treatments.

In contrast, the neutralization of IL-1 abrogated the therapeutic effect of chemotherapy [66], pointing to the possibility that according to the nature of the cancer and of the TME, the blockage of the inflammasome could open new perspectives for the treatment of cancer. Noteworthy, the overall consequences of inflammasome activation and IL-1β release following chemotherapeutic treatment may vary with the stage of the tumor. The early production of IL-1β may be advantageous according to the immunogenic anti-tumor response mediated by the innate and adaptive immune system against dying tumor cells [27]; instead, at advanced stages, an IL-1β-dependent response may contribute to tumor growth, limiting the anti-tumor efficacy of chemotherapeutic agents [8,47].

From this prospective, an interventional study was projected in order to evaluate the safety of Anakinra plus standard chemotherapy (oxaliplatin + irinotecan + 5-FU) in metastatic pancreatic ductal adenocarcinoma (PDCA) [67]. The results showed that Anakinra in combination with standard chemotherapy is safe and feasible [68].

In line with that, a study conducted on malignant mesothelioma (MM) cells proposes that combining chemotherapeutic drugs with IL-1 receptor antagonist may have a beneficial effect on MM tumor reduction [13]; specifically, treatment with cisplatin or doxorubicin induced NLRP3 inflammasome priming and activation, both an anti-tumorigenic effect (through caspase-1-dependent cell death) and pro-tumor effect (through enhanced IL-1β, IL-18, fibroblast growth factor 2 (FGF2), and HMGB1 release) ensuing. It is conceivable that the initial increase in IL-1β levels in response to chemotherapeutic drugs could be sufficient to start inflammasome-dependent signaling cascades so that the usage of interleukin-1 receptor antagonist (IL-1Ra; Anakinra) before chemotherapy could be a better strategy for MM tumor treatment [12]. Even more, Antonopoulos et al. [69] suggest a new pathway involving a caspase-8-dependent non-canonical inflammasome for IL-1β release following treatment with proapoptotic chemotherapeutic drugs [69]. In this study, the co-stimulation of bone marrow-derived DCs (BMDCs) with TLR4 agonists (i.e., lipopolysaccharide, LPS) and pro-apoptotic agents such as doxorubicin or staurosporine, induced IL-1β processing by caspase-8 signaling, indicating the engagement of an alternative regulated cell death pathway [69].

While information on the anti-cancer role of NLRP3, nothing is known regarding AIM2 inflammasome. To our knowledge, few studies support the beneficial effect dependent on AIM2 after chemotherapy treatment. One research hypothesizes that Tumor Treating Fields (TTFields), approved in combination with adjuvant temozolomide chemotherapy for newly diagnosed glioblastoma (GBM) patients, induced a significant improvement in overall survival after AIM2 and cGAS activation [70]. Specifically, according to the authors, TTFields activate the immune system by triggering AIM2-dependent pyroptosis and STING-dependent-type I Interferon (IFN) response, tumor suppression ensuing [70]. In support, the double knock down of STING/AIM2 in a mouse model of GBM, eliminated the tumor immune-suppression effects caused by TTFields [70]. Another study highlights that low-dose chemotherapy preferentially leads to DNA damage and the accumulation of cytosolic dsDNA in ileal epithelial cells, able to activate the AIM2 inflammasome signaling to trigger cell–cell crosstalk in the small intestine [16]; specifically, the authors found that AIM2-dependent IL-18 release led to the interplay between proximal Th1 and Paneth cells in ileal crypts responsible of the impairment of antimicrobial functions of proximal Paneth cells and the ensuing ileal microbiome changes associated with anti-tumor immune response [16]. Moreover, chemotherapy-induced AIM2 activation was associated with increased anti-tumor responses to anti-programmed cell death protein 1 (PD-1) therapy.

**Table 1:** Inflammasome-targeting chemotherapeutic agents and their effects. This table summarizes chemotherapeutic and immunomodulating agents targeting the inflammasome pathway, their biological effect, and their impact on cancer-associated inflammation and tumor growth.

Therapeutic Agent(s)	Inflammasome Target or Biological Effect	Impact on Inflammation and Cancer	Experimental Approaches	References
P2X7R antagonists	Inhibition of P2X7 pathway	Reduction of tumor proliferation and invasion in pancreatic ductal adenocarcinoma and of invasiveness in epidermoid carcinoma	In vitro studies	[30,31]
Polyclonal anti-nfP2X7 antibodies (BIL010t)	Blockade of nfP2X7 receptor, a variant of P2X7 in which the E200 epitope is exposed on the surface of tumor cells	Decrease in basal cell carcinoma lesion area	phase I clinical trial	[32]
Paclitaxel	TLR4 signaling and NLRP3 priming	Possible limitation of drug efficacy	In vivo and ex vivo studies	[14]
Gemcitabine and 5-FU	NLRP3 inflammasome activation and IL-1β release	Limitation of drugs anti-tumor efficacy		[47]
5-FU	Release of intracellular ROS and the following NLRP3 inflammasome activation	Poor prognosis and chemoresistance in oral squamous cell carcinoma	In vivo and ex vivo studies	[57]
Anthracyclines	ICD induction with ensuing release of IL-1β and priming of anti-cancer CD8^+^ T cell	Acute inflammation associated with beneficial for anti-cancer responses	In vitro studies	[55]
DHA	Inhibition of 5-FU-induced NLRP3 inflammasome activation	Possible improvement in 5-FU efficacy in colorectal cancer	In vivo and ex vivo studies	[54]
Cisplatin or Doxorubicin	NLRP3 inflammasome priming and activation	Anti-tumor effect through caspase-activation in malignant mesothelioma	In vitro studies	[13]
Anakinra	IL-1R inhibition which counteracts the initial increase in IL-1β levels in response to chemotherapeutic drugs	Improvement in Cisplatin or Doxorubicin efficacy in malignant mesothelioma	In vivo studies	[13]
Anakinra	IL-1β blockade and reduction of IL-6 levels in myeloma cells	Prolongs the progression-free survival of indolent myeloma patients	phase II clinical trial	[62]
Anakinra + Dexamethasone	IL-1R inhibition and immunosuppression	Decrease of myeloma cells proliferation	phase II clinical trial	[64]
Anakinra + 5-FU + Bevacizumab	IL-1R inhibition + NLRP3 inflammasome activation + VEGF blockade	Improvement in median progression-free and overall survival in metastatic colorectal cancer patients	phase II clinical trial	[65]
Thalidomide	Inhibition of caspase-1, associated with inflammasome activation	Increase in the median survival rate of malignant myeloma patients and increase of Docetaxel efficacy in metastatic androgen-independent prostate cancer	phase II clinical trial	[58,60]

Abbreviations: P2X7R, P2X7 receptor; TLR4, Toll-like receptor 4; NLRP3, NOD-like receptor family pyrin domain-containing-3; 5-FU, 5-fluorouracil; ROS, reactive oxygen species; ICD, immunogenic cell death, DHA, docosahexaenoic acid; IL-1R, Interleukin 1 receptor; VEGF, vascular endothelial growth factor.

## 4. Conclusions

Cancer-associated inflammatory responses play a critical role in many aspects of cancer biology, including tumor initiation, progression, metastasis, and treatment. In this context, the inflammasome involvement represents a double-edged sword in that it still remains to be understood if its activation during the neoplastic transformation/cells proliferation or pharmacological treatment can be beneficial or not for the oncological patient (Table 1). In our opinion, the answer to this question underlies the nature and the tumor microenvironment. Preclinical studies have revealed that the activation of the inflammasome can have dual activity according to the experimental approach and treatment. In particular, the treatment of tumors with conventional chemotherapy can facilitate the release of DAMPs that, via ICD, can promote in situ inflammation able to either boost the immune system or contribute to the tolerogenic and immunosuppressant environment. In the latter case, we believe that the immune-suppressive arm overcomes it, such as during a physiological response that tries to downregulate an excessive immune response that could be detrimental for the host. Thus, the activation of the inflammasome and the ensuing immune cells after chemotherapy-induced ICD still needs to be elucidated in detail according to the stage/histotype of cancer as well as the pharmacological treatment.
